# Reliability and Validity of the Transport and Physical Activity Questionnaire (TPAQ) for Assessing Physical Activity Behaviour

**DOI:** 10.1371/journal.pone.0107039

**Published:** 2014-09-12

**Authors:** Emma J. Adams, Mary Goad, Shannon Sahlqvist, Fiona C. Bull, Ashley R. Cooper, David Ogilvie, on behalf of the iConnect Consortium

**Affiliations:** 1 British Heart Foundation National Centre for Physical Activity and Health, School of Sport, Exercise and Health Sciences, Loughborough University, Loughborough, United Kingdom; 2 Centre for Physical Activity and Nutrition Research (C-PAN), School of Exercise and Nutrition Sciences, Deakin University, Geelong, Australia; 3 Centre for the Built Environment and Health, School of Population Health, University of Western Australia, Perth, Australia; 4 Centre for Exercise, Nutrition and Health Sciences, School for Policy Studies, University of Bristol, Bristol, United Kingdom; 5 Medical Research Council Epidemiology Unit and UKCRC Centre for Diet and Activity Research (CEDAR), University of Cambridge, Cambridge, United Kingdom; Texas A&M University, United States of America

## Abstract

**Background:**

No current validated survey instrument allows a comprehensive assessment of both physical activity and travel behaviours for use in interdisciplinary research on walking and cycling. This study reports on the test-retest reliability and validity of physical activity measures in the transport and physical activity questionnaire (TPAQ).

**Methods:**

The TPAQ assesses time spent in different domains of physical activity and using different modes of transport for five journey purposes. Test-retest reliability of eight physical activity summary variables was assessed using intra-class correlation coefficients (ICC) and Kappa scores for continuous and categorical variables respectively. In a separate study, the validity of three survey-reported physical activity summary variables was assessed by computing Spearman correlation coefficients using accelerometer-derived reference measures. The Bland-Altman technique was used to determine the absolute validity of survey-reported time spent in moderate-to-vigorous physical activity (MVPA).

**Results:**

In the reliability study, ICC for time spent in different domains of physical activity ranged from fair to substantial for walking for transport (ICC = 0.59), cycling for transport (ICC = 0.61), walking for recreation (ICC = 0.48), cycling for recreation (ICC = 0.35), moderate leisure-time physical activity (ICC = 0.47), vigorous leisure-time physical activity (ICC = 0.63), and total physical activity (ICC = 0.56). The proportion of participants estimated to meet physical activity guidelines showed acceptable reliability (k = 0.60). In the validity study, comparison of survey-reported and accelerometer-derived time spent in physical activity showed strong agreement for vigorous physical activity (r = 0.72, p<0.001), fair but non-significant agreement for moderate physical activity (r = 0.24, p = 0.09) and fair agreement for MVPA (r = 0.27, p = 0.05). Bland-Altman analysis showed a mean overestimation of MVPA of 87.6 min/week (p = 0.02) (95% limits of agreement −447.1 to +622.3 min/week).

**Conclusion:**

The TPAQ provides a more comprehensive assessment of physical activity and travel behaviours and may be suitable for wider use. Its physical activity summary measures have comparable reliability and validity to those of similar existing questionnaires.

## Introduction

Current approaches for increasing population levels of physical activity include promoting walking and cycling for transport and recreation. This has the potential to support policy goals in a number of sectors including public health (increasing physical activity levels), transport (increasing the use of sustainable travel modes) and environment (reducing carbon emissions) [1]–[3]. Increasingly, interdisciplinary research teams are working together to advance research in this area and require comprehensive measures of physical activity and travel behaviours to meet their different needs [3]–[5]. These measures need to enable assessment of the frequency and duration of participation in specific domains of physical activity, the total amount of physical activity undertaken, and the time spent and distance travelled using different modes of motorised and non-motorised transport for specific journey purposes. To date, no such comprehensive instruments have been developed and tested for their reliability and validity. A few instruments do cover some of these items, but these generally omit certain domains of activity (such as recreational activity), journeys made for certain purposes or the use of modes of transport other than walking and cycling [6], [7]. In some cases the use of modes of transport other than walking and cycling is measured but the modes are grouped collectively under the term ‘motorised vehicles’, as in the long version of the International Physical Activity Questionnaire (IPAQ) [8]. This means it is not possible to assess the use of individual modes of motorised travel, such as car, train or bus, separately.

The development of a comprehensive instrument presents a challenge because of differences in the measures and approaches used by each discipline for assessing the different behaviours of interest. Physical activity research in adults typically uses self-report questionnaires or telephone surveys which ask about physical activity participation in different domains in the previous week or month [9]. The reliability and validity of instruments of this kind, such as the IPAQ [8], Global Physical Activity Questionnaire (GPAQ) [10] and Neighbourhood Physical Activity Questionnaire (NPAQ) [11], are usually tested and reported. However, in the transport sector, trip diaries and intercept surveys are more commonly used and the measurement properties of these instruments are often not tested or reported [12].

Although many physical activity questionnaires already exist and have been tested for reliability and validity, these instruments are often amended by researchers, who have slightly differing needs for their specific research project, without further reliability and validity testing. The effect of making these small changes on reliability and validity is largely unknown which raises a question as to whether further reliability and validity studies are needed each time an existing survey is adapted or whether it is safe to assume that the measurement properties are likely to remain similar.

The current study was conducted within the iConnect (Impact of COnstructing Non-motorised Networks and Evaluating Changes in Travel) project, a five year natural experimental study to assess the impact of improving walking and cycling infrastructure on travel, physical activity and carbon emissions [13]. In view of the lack of suitable existing instruments for assessing physical activity, walking and cycling for recreation, walking and cycling for transport and detailed measures of travel behaviour using other modes, a new transport and physical activity questionnaire (TPAQ) was developed. This paper reports on the development of the TPAQ, the test-retest reliability and validity of the physical activity items in the TPAQ and the impact of modifying an existing instrument, the IPAQ [8].

## Methods

### 

#### Development and content of the TPAQ

The TPAQ was developed by an interdisciplinary consortium as part of the iConnect study in the UK. Full details of the study, including a freely downloadable copy of the survey which incorporates the TPAQ, are provided elsewhere [14]. The development phase included a feasibility study [15] and pilot reliability and validity studies (not reported). The TPAQ was designed to enable a detailed assessment of time spent and distance travelled using different modes of transport overall and for different journey purposes, as well as time spent in different domains of recreational physical activity. First, travel behaviour in the last seven days was assessed across five categories of trip purpose: 1. to and from work, 2. for business purposes, 3. to and from a place of study, 4. for shopping and personal business, and 5. to visit friends or family or for other social activities. For each journey purpose participants were asked to report the number of trips, the total time spent (in hours and minutes) and the total distance (in miles) travelled in the last seven days for each of six specified modes of transport (walking, cycling, bus, train, car as a driver and car as a passenger) or any ‘other’ mode (which captured modes such as taxi and van). Four domains of activity (walking for recreation, cycling for recreation, moderate leisure-time activity and vigorous leisure-time activity) were assessed using items adapted from the short form of the IPAQ [8]. The questions asked participants to report the number of sessions and the total duration of participation (in hours and minutes) in each of the four domains of activity in the last seven days excluding any activity they had previously reported. The items were then used to create a number of key physical activity summary variables (Table 1). In addition the survey asked respondents to report their sex, age, ethnic group, educational qualifications, housing tenure, and the number of cars and bicycles in the household. This paper reports on the reliability and validity of the physical activity summary variables measured using the TPAQ. The reliability and validity of the travel behaviour measures will be reported separately.

**Table 1:** Questionnaire items and physical activity summary variables in TPAQ.

Questionnaire item	Response	Items included in physical activity summary variables									
		Reliability study							Validity study		
TRAVEL BEHAVIOUR		Wt^r^	Ct^r^	Wr^r^	Cr^r^	MPA^r^	VPA^r^	TPA^r^	MPA^v^	VPA^v^	MVPA^v^
**For each of five journey purposes^†^:**											
How often did you make such a journey over the last 7 days?	Number of times										
How much time in total over the last 7 days did you spend travelling to and from work by:	Hours/minutes spent:										
	Walking	✓♦						✓♦	✓♦		✓♦
	Cycle		✓♦					✓♦			
	Bus										
	Train										
	Car, as a driver										
	Car, as a passenger										
	Other (specify)										
**PHYSICAL ACTIVITY**											
In the last 7 days, how many times did you **walk** for recreation, health or fitness (including walking your dog)?	Number of times			✓^f^							
Please estimate the total time you spent **walking** for recreation, health or fitness in the last 7 days.	Hours Minutes			✓				✓	✓		✓
In the last 7 days, how many times did you **cycle** for recreation, health or fitness?	Number of times				✓ f						
Please estimate the total time you spent **cycling** for recreation, health or fitness in the last 7 days.	Hours Minutes				✓			✓			
During the last 7 days, how many times did you do **moderate intensity leisure time** physical activities which make you breathe somewhat harder than normal?	Number of times					✓ f					
What do you estimate is the total time you spent doing **moderate intensity leisure time** physical activities in the last 7 days?	Hours Minutes					✓		✓	✓		✓
During the last 7 days, how many times did you do **vigorous intensity leisure time** physical activities which makes you breathe harder or puff and pant?	Number of times						✓ f				
What do you estimate is the total time you spent doing **vigorous intensity leisure time** physical activities in the last 7 days?	Hours Minutes						✓	✓		✓	✓

†Journey purposes: to and from work; business journeys; to and from a place of study or to and from school; for shopping and personal business; to visit friends and relatives and for other social activities. Wt = walking for transport; Ct = cycling for transport; Wr = Walking for recreation; Cr = cycling for recreation; VPA = vigorous physical activity; MPA = moderate physical activity; TPA = Total physical activity; MPA = moderate physical activity; TPA = Total physical activity; MVPA = moderate-to-vigorous physical activity.

r = reliability study;

v = validity study;

f = frequency of participation assessed in reliability study as separate variable;

♦summed across all journey purposes. The full survey is available at http://bmjopen.bmj.com/content/suppl/2012/02/04/bmjopen-2011-000694.DC1/bmjopen-2011-000694-s1.pdf

### Ethics statement

The reliability and validity studies received ethical approval from the University of Southampton Ethics Committee (CEE 200809-15).

### Reliability study

#### Participants and procedures

In October 2010, 3000 adults were randomly selected from the edited electoral register for six wards in the town of Loughborough, UK and invited to complete the iConnect survey on two separate occasions, approximately seven days apart. The initial mailing contained a letter of invitation, a copy of the survey, a consent form and a freepost return envelope. Individuals who completed and returned the first survey (n = 216) were then posted the second survey and asked to complete and return it as soon as possible. Participants who did not return the second survey within seven days received a reminder phone call or letter. A prize draw to win one of twenty £25 gift vouchers was offered for those who completed both surveys as an incentive for participation.

#### Physical activity summary variables

Seven continuous physical activity summary variables (number of minutes spent in the past week in walking for transport, cycling for transport, walking for recreation, cycling for recreation, moderate leisure-time physical activity, vigorous leisure-time physical activity and total physical activity) and one categorical variable (proportion meeting current UK physical activity guidelines) were computed. Time spent walking for transport for each of the five journey purposes was summed to give the total time spent walking for transport. A similar process was followed for total time spent cycling for transport. A measure of total physical activity was computed by summing time spent walking and cycling for transport, time spent walking and cycling for recreation and time spent in moderate and vigorous leisure-time physical activity. This measure was also used to derive a categorical variable indicating whether respondents met the level of physical activity for adults recommended in the 2011 UK guidelines of at least 150 minutes of moderate intensity activity per week [16].

#### Analyses

The test-retest reliability of the seven continuous physical activity summary variables and the categorical variable was assessed. In secondary analyses, the reliability of the frequency of participation (number of sessions per week) was assessed for walking for recreation, cycling for recreation, moderate leisure-time physical activity and vigorous leisure-time physical activity.

Descriptive results are reported as mean ± standard deviation (SD). Intra-class correlation coefficients (ICC) were used to assess the reliability of each of the continuous variables and Kappa scores were used to assess the reliability of the categorical variable. For the purposes of this study, coefficient values of ≤0.20 were taken to indicate poor agreement; 0.21–0.40 represented fair agreement; 0.41–0.60 represented moderate or acceptable agreement; 0.61–0.80 represented substantial agreement; and 0.81–1.0 represented almost perfect agreement [17].

### Validity study

#### Participants and procedures

The validity study was conducted between June and October 2011. Participants who took part in the reliability study and who had agreed to take part in future studies (n = 136) were sent a letter inviting them to take part in the validity study. In addition, employees from Loughborough University and three local small-to-medium-sized businesses were invited to take part in the project via email and word of mouth.

Individuals who registered their interest in participating in the study were invited to attend a group meeting where they were provided with an accelerometer (Actigraph GT3X, Actigraph, Pensacola, Florida, USA), a global positioning system (GPS) data logger (QSTARZ BT-Q1000) and a copy of the iConnect survey. A researcher explained the study to participants, including how to use the accelerometer and GPS device and when to complete the survey, and obtained written consent from each participant. Participants were instructed to wear the accelerometer and GPS device around their waist on the right hip for seven consecutive days from getting out of bed in the morning until going to bed at night, except when participating in water-based activities. Participants were asked to complete the survey on the eighth day. To maximise adherence to the study protocol, on the third day of monitoring participants received a follow-up telephone call or email to resolve any issues or concerns about the study. Participants were also contacted on day seven to remind them to complete the survey the following day and to arrange a time and place for the researcher to collect the study materials. Each participant in the validity study received a £5 gift voucher.

This study reports on the validity of the survey-derived physical activity summary variables using accelerometer data. GPS data were used only for the validation of the travel behaviour measures, which will be reported separately.

#### Data processing and accelerometer-derived physical activity summary variables

Accelerometers were programmed to record data at ten second epochs. Raw accelerometer data were processed using MAHUffe (MRC Epidemiology Unit, Cambridge, http://www.mrc-epid.cam.ac.uk). The last day of recording (the day on which the accelerometer was collected from the participant) and continuous periods where 60 or more minutes of zero values were recorded were considered to be non-wear time and were excluded. To maximise study quality we applied a strict inclusion criterion whereby participants were only included in analyses if they had worn the accelerometer for six or more days for at least 10 hours per day and had completed the survey within 2 days of their last day of accelerometry (n = 54). For participants with only six days of objectively measured physical activity (n = 9), the mean values for time spent in physical activity across the six recorded days were calculated and added to the six day total to estimate a value for seven days, the time period required for comparison with the survey data. Data were aggregated into 60 second epochs and cut points were used to classify data into different intensity activities: sedentary (0–199 counts per minute (cpm)), light intensity activity (200–2019 cpm), moderate intensity activity (2020–5998 cpm), or vigorous/very vigorous intensity activity (>5998 cpm) [18], [19]. Summary variables were then computed representing time spent in moderate physical activity; time spent in vigorous physical activity (includes vigorous/very vigorous intensity activity) and time spent in moderate-to-vigorous physical activity (MVPA) (sum of time spent in moderate and vigorous physical activity).

#### Survey-derived physical activity summary variables

Three physical activity summary variables (moderate physical activity, vigorous physical activity and MVPA) were computed using survey data for comparison with accelerometer data. Times spent walking for transport, walking for recreation and in moderate leisure-time physical activity were summed to give time spent in moderate physical activity. Self-reported time spent in vigorous leisure-time physical activity was used for comparison with accelerometer-derived time spent in vigorous activity. A measure of total MVPA was computed by summing time spent walking for transport, time spent walking for recreation and time spent in moderate and vigorous leisure-time physical activity. Cycling was excluded from the survey-derived physical activity summary variables used in the validity study because hip-worn accelerometers have limited capacity to detect cycling [20] and time spent cycling has been shown to be a significant contributor to the disagreement between self-reported and objectively measured estimates of activity [21]. A sensitivity analysis was, however, conducted to determine whether this had any impact on the results (see below).

#### Analyses

Descriptive statistics are reported as mean ± standard deviation (SD). Correlation coefficients were calculated for survey-reported and accelerometer-derived summary physical activity variables using Spearman's rho because the data were not normally distributed.

Absolute validity was assessed by determining the agreement between self-reported total time spent in MVPA and accelerometer-derived total time spent in MVPA using the Bland-Altman technique [22]. A mean bias was defined as a significant mean difference obtained by subtracting objectively measured time spent in physical activity from self-reported time spent in physical activity. The error was defined as 2 SD of the mean bias.

Analyses for the reliability and validity studies were conducted using Statistical Package of Social Sciences, version 19.0.0 for Windows (IBM SPSS Inc, Chicago, Illinois, USA). Bland-Altman analyses were conducted using STATA 13 (StataCorp LP, College Station, Texas, USA).

#### Sensitivity analysis

A sensitivity analysis was conducted to determine the impact of including cycling in the survey-derived data for the analyses. Additionally, because participants may have reported transport or recreational walking undertaken at light intensity (<3 METs) [23] we conducted a second sensitivity analysis in which time spent in objectively-measured light intensity activity was included in the accelerometer-derived summary variables for total physical activity and moderate intensity physical activity. For both analyses, correlation co-efficients were calculated and absolute validity was assessed.

## Results

### Reliability study

Of the 3000 adults invited to take part in the reliability study, 166 completed and returned both surveys (a response rate of 6%) and were included in the analysis (Table 2).

**Table 2:** Sample characteristics.

Characteristic		Reliability study		Validity study	
		n = 166		n = 54^a^	
		n^b^	%	n^b^	%
**Sex**	Male	77	46.7	19	35.2
**Age (years)**	<30	19	11.7	12	22.2
	30–44	40	24.5	13	24.1
	45–64	59	36.2	25	46.3
	≥65	45	27.6	4	7.4
**Ethnicity**	White	147	90.7	53	98.1
	Other	15	9.3	1	1.9
**Education**	Degree	51	31.7	35	64.8
	GCE ‘A’ Level	28	17.4	10	18.5
	GCSE Grades A to C	39	24.2	5	9.3
	No formal qualification	43	26.7	4	7.4
**Housing tenure**	Owned	133	81.1	44	81.5
	Rented from private landlord	10	6.1	9	16.7
	Rented from local authority	19	11.6	1	1.9
	Other	2	1.2	0	0.0
**Household cars**	0	20	12.3	6	11.1
	1	71	43.8	18	33.3
	2 or more	71	43.8	30	55.6
**Household bicycles**	0	57	34.3	7	13.0
	1 more	109	65.7	47	87.0

a Included in analysis.

b Numbers do not sum to totals due to missing responses.

For each of the physical activity summary variables the mean weekly duration of physical activity was lower at the second survey administration (T2), which took place on average 12.4±6.6 days after the first survey administration (T1) (Table 3). The reliability of the survey-based time measures differed according to the specific activities being assessed. For transport-related activity, the intra-class correlation (ICC) of items was substantial for time spent cycling for transport (ICC = 0.61) and acceptable for time spent walking for transport (ICC = 0.59). For recreational activities, there was acceptable agreement for time spent walking for recreation (ICC = 0.48) but only fair agreement for time spent cycling for recreation (ICC = 0.35). Agreement was substantial for time spent in vigorous leisure-time physical activity (ICC = 0.63) and acceptable for time spent in moderate leisure-time intensity physical activity (ICC = 0.47) (Table 3). There was acceptable agreement between the two survey administrations for total duration of physical activity (ICC = 0.56) (Table 3) and for the proportion of respondents meeting physical activity recommendations (Kappa = 0.60) (data not shown).

**Table 3:** Intra-class correlations for time spent in transport-related and recreational physical activity overall and by domain.

Physical activity domain		T1	T2	
	n	Mean minutes in last week ±SD	Mean minutes in last week ±SD	ICC (95% CI)
Walking for transport	164	142.0±256.6	129.6±194.4	0.59 (0.48, 0.68)
Cycling for transport	164	25.7±92.6	21.5±92.6	0.61 (0.50, 0.70)
Walking for recreation	165	117.1±192.2	116.6±207.4	0.48 (0.35, 0.59)
Cycling for recreation	165	27.4±104.6	20.4±105.8	0.35 (0.20, 0.47)
Moderate intensity leisure-time physical activity	165	80.7±168.4	63.2±131.8	0.47 (0.34, 0.58)
Vigorous intensity leisure-time physical activity	163	60.6±134.2	49.8±97.1	0.63 (0.53, 0.71)
Total physical activity†	161	441.2±455.3	387.4±374.0	0.56 (0.45, 0.66)

T1 = Survey time point 1;

T2 = Survey time point 2;

† Total physical activity includes vigorous and moderate leisure-time activity and walking and cycling for transport and recreation.

For frequency of participation, there was substantial agreement for the number of sessions of walking (ICC = 0.80) and cycling (ICC = 0.63) for recreation, acceptable agreement for the number of sessions of vigorous intensity leisure-time physical activity (ICC = 0.52) and poor agreement for the number of sessions of moderate intensity leisure-time physical activity (ICC = 0.13) (Table 4).

**Table 4:** Intra-class correlations for frequency of participation in recreational physical activity.

Physical activity domain		T1	T2	
	n	Mean times in last week (±SD)	Mean times in last week (±SD)	ICC (95% CI)
Walking for recreation	165	2.6±4.2	2.4±4.1	0.80 (0.73, 0.85)
Cycling for recreation	165	0.4±1.3	0.4±1.2	0.63 (0.53, 0.71)
Moderate intensity leisure-time physical activity	166	1.0±1.7	0.8±2.4	0.13 (−0.02, 0.28)
Vigorous intensity leisure-time physical activity	166	1.1±1.9	0.9±1.8	0.52 (0.40, 0.62)

T1 = Survey time point 1;

T2 = Survey time point 2.

### Validity study

A total of 72 participants took part in the validity study. These were recruited from among those who took part in the reliability study (n = 25; a response rate of 18%) and from the convenience samples of staff and students of Loughborough University (n = 27) and local small-to-medium-sized businesses (n = 20). Of those who took part in the validity study 54 participants were eligible for inclusion in the analysis (Table 2).

In comparison to objectively-measured data, participants tended to over-report moderate and vigorous physical activity which resulted in an over-reporting of total physical activity in the survey (Table 5). Survey-reported moderate physical activity (including moderate leisure-time physical activity, walking for transport and walking for recreation, but excluding any cycling) showed fair agreement with objectively measured time spent in moderate physical activity (r = 0.24, p = 0.085). In contrast, survey-reported vigorous physical activity showed strong agreement with objectively-measured vigorous physical activity (r = 0.72, p<0.001), although this relatively high correlation concealed large differences in the absolute estimates in that the mean duration of survey-reported vigorous physical activity (99.2 min/week) was more than three times higher than that of objectively-measured vigorous physical activity (30.2 min/week). Agreement between survey-reported MVPA and objectively-measured MVPA was fair and of borderline statistical significance (r = 0.27, p = 0.051) (Table 5).

**Table 5:** Validity of physical activity measures overall and by domain.

Physical activity domain		Survey items included in summary variable	Accelerometer-derived data included in summary variable	Survey data	Accelerometer data		
	n			Mean minutes (±SD)	Mean minutes (±SD)	r	p
Moderate physical activity	53	Moderate intensity leisure-time physical activity	Moderate intensity physical activity	307.9±227.2	294.5±133.5		
		Walking for transport					
		Walking for recreation					
Vigorous physical activity	46	Vigorous intensity leisure-time physical activity	Vigorous intensity physical activity	99.1±141.6	30.2±67.5	0.72	<.001
Moderate-to-vigorous physical activity	52	Vigorous intensity leisure-time physical activity	Vigorous intensity physical activity	431.1±256.7	343.5±164.4	0.27	.051
		Moderate intensity leisure-time physical activity	Moderate intensity physical activity				
		Walking for transport					
		Walking for recreation					

In sensitivity analysis, including light intensity activity in the accelerometer-derived summary variables reduced the agreement between self-reported and objectively-measured moderate physical activity (r = 0.17, p = 0.226) whereas the agreement for MVPA improved slightly and became significant (r = 0.29, p = 0.037). Including cycling in the survey-derived summary variables reduced the agreement between survey-reported and objectively-measured moderate physical activity (r = 0.10, p = 0.495) and between survey-reported and objectively-measured MVPA (r = 0.09, p = 0.518) (data not shown).

**Figure 1:**
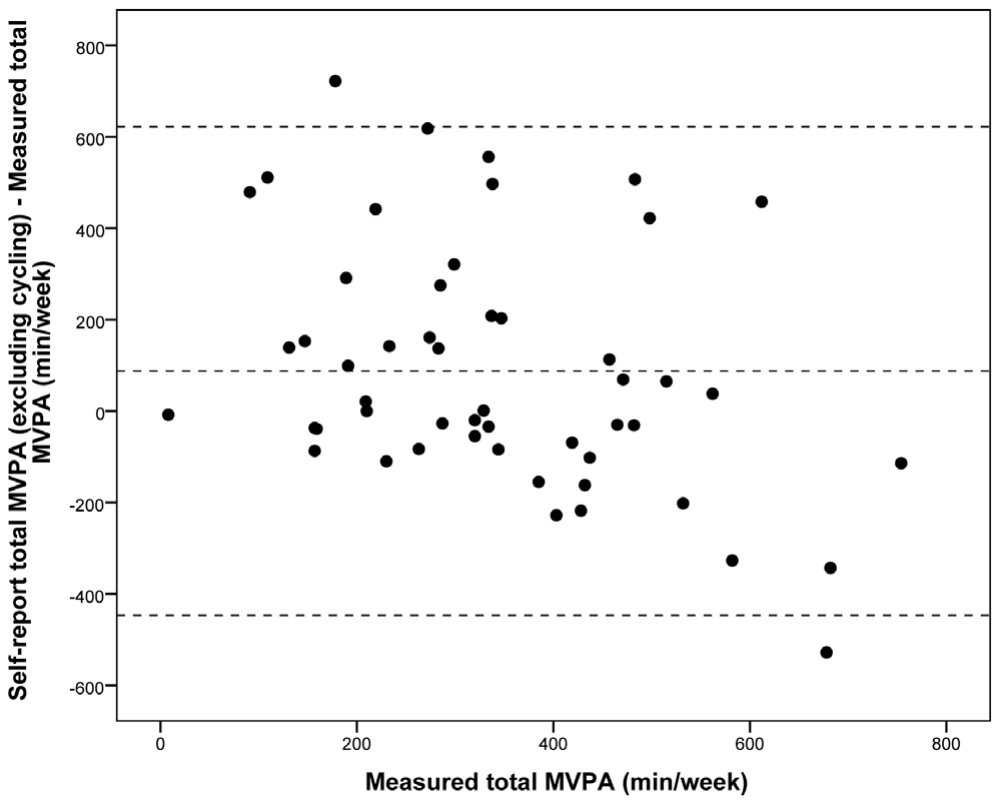
Bland-Altman plot for time spent in moderate-to-vigorous physical activity. The difference between objectively measured time spent in total MVPA (min/week) and self-reported time spent in MVPA excluding cycling (min/week) plotted against objectively measured time spent in MVPA (min/week). Mean difference: 87.6 min/week (p = 0.02); limits of agreement: −447.1 min/week, +622.3 min/week.

A Bland-Altman plot for the main analysis is shown in Figure 1. The difference between self-reported and accelerometer-derived MVPA for each participant is plotted on the *y* axis against their accelerometer-derived MVPA on the *x* axis. A mean overestimation of self-reported MVPA of 87.6 min/week (p = 0.02) was observed and the 95% limits of agreement were wide (−447.1 to +622.3 min/week).

In sensitivity analysis including light intensity accelerometer activity, the mean difference was greater at −1407.5 min/week (p<0.001) with commensurately wider 95% limits of agreement (−2314.4 to −500.6 min/week). In sensitivity analysis including cycling for transport and recreation in survey-reported total physical activity, the mean difference was 202.4 min/week (p<0.001) with 95% limits of agreement of −473.6 to +878.3 min/week (data not shown).

## Discussion

A new instrument (the TPAQ) was developed for use in interdisciplinary research on physical activity and travel behaviours. The instrument allows specific target behaviours (walking and cycling separately and for different purposes) to be measured as well as providing an estimate of total physical activity. Developing such instruments is challenging given the different approaches typically used by different disciplines to assess the behaviours of interest and differences in terminology applied to similar constructs.

### Reliability and validity of physical activity summary variables derived from TPAQ

TPAQ, which combines new items on travel behaviour with an adapted version of the short form of IPAQ, appears to be an acceptably reliable measure of time spent in different domains of physical activity as well as total physical activity. In most respects its reliability was comparable to that of other similar instruments used for measuring physical activity [8], [10], [11], [24], [25]. We used the last seven days as the reference period, which may have resulted in lower stability in our measures of behaviours such as walking and cycling for transport and recreation than would have been observed using an instrument framed in terms of a ‘usual’ week.

The reported frequency of participation in most types of leisure-time physical activity (walking for recreation, cycling for recreation and vigorous intensity physical activity) showed greater reliability than that for the reported duration of activity. Similar findings have been reported elsewhere [11], raising the question of whether it may be more appropriate to assess frequency of participation than to assess duration. However, the test-retest reliability of reported frequency of moderate intensity physical activity was poor, and it is more difficult to impute health benefits to a measure of frequency of participation than to an estimate of total volume (e.g. duration) of activity.

Compared to an accelerometer-derived criterion, TPAQ produced an estimate of total time spent in physical activity of comparable validity to that of the short IPAQ [8], [25] and most other off-the-shelf questionnaires of similar length currently in use. In Bland-Altman analysis, TPAQ significantly overestimated MVPA in comparison to accelerometer-derived estimates by 88 minutes per week, with no clear pattern of any change in bias with higher levels of self-reported physical activity. Other studies have also reported an overestimation of self-reported MVPA in comparison to accelerometer-measured MVPA, although these differences have been much larger than those observed in our study. For example, one recent study found an overestimation of 46 minutes per day between GPAQ and objectively-measured MVPA, and 76 minutes per day between short IPAQ and objectively-measured MVPA [26]. The observed limits of agreement in our study were very wide (over 10 hours per week) however other studies comparing self-reported MVPA with objectively-measured MVPA have reported even larger limits of agreement, for example up to 17 hours per week using long IPAQ [27] or two hours per day using short IPAQ [28]. We therefore conclude that TPAQ is as acceptable as other similar instruments for use in measuring MVPA, notwithstanding the widely recognised limitations of all self-reported estimates of physical activity.

The substantial agreement observed for vigorous physical activity estimated using the TPAQ is higher than that reported elsewhere [25]. However it appears that the TPAQ may overestimate the time spent in this domain of activity and thus overall physical activity. The poor agreement between survey-reported and accelerometer-measured moderate physical activity has also been observed in a number of other studies [25]. This may reflect participants having reported light intensity activities in the survey, as TPAQ does not specify the intensity of walking and cycling for transport or recreation that should be reported. Participants may therefore have reported walking or cycling of any intensity. Including light intensity accelerometer-derived activity in a sensitivity analysis did improve the agreement slightly for total physical activity. These findings suggest that the performance of TPAQ as a measure of moderate and vigorous physical activity could be improved by specifying the intensity of walking and cycling that should be reported and more clearly defining which activities should be reported as being of vigorous intensity. The inclusion of cycling in survey-reported measures reduced the agreement with accelerometer-derived variables, supporting previous findings that time spent cycling may contribute to the disagreement between self-reported and objectively measured estimates of activity [21].

TPAQ asks about participation in a number of different domains of physical activity in order to provide more specific outcome measures for particular interventions that might target, for example, walking or cycling for transport or recreation. In the validity study, this appeared to lead to an over-reporting of physical activity compared to accelerometer-derived estimates. It is not yet clear whether disaggregating physical activity behaviours in this way leads to over-reporting, or whether it increases accuracy because participants can recall the time they have spent in more specific activities more precisely than they can provide a more global estimate of time spent in overall physical activity. Other possible reasons for the higher estimates from self-report data include social desirability bias which may have led to participants over-reporting their activity; difficulties in recalling activities over the past week; inclusion of short (<10 minute) bouts of activity; or the need for participants to sum time spent in different activities over the previous week. High estimates may also be due to participants reporting walking or cycling under both ‘transport’ and ‘recreation, health or fitness’ headings, because it is sometimes difficult to distinguish these purposes.

### Adaptation of physical activity items from existing instruments

Instruments used to assess physical activity behaviour are often adapted by investigators for use in their research projects without further reliability and validity testing. In developing the TPAQ, recreational and leisure-time physical activity items were adapted from an existing instrument, the short IPAQ. We found the physical activity summary variables derived from TPAQ to have broadly comparable reliability and validity to those of the original IPAQ [8], suggesting that minor modifications to survey instruments may not necessarily alter their reliability and validity to the extent that their measurement properties would always need to be reassessed. However, IPAQ itself has been tested in a large number of different populations from different countries [8], [9], [25] with varying findings, suggesting that it is not only the wording of the surveys but also the target group and context in which they are used that may affect reliability and validity.

### Strengths and limitations

We successfully developed an instrument to meet the needs of interdisciplinary research on physical activity and travel behaviour. We tested its reliability and validity predominantly using a community sample rather than a convenience sample, which helps to improve the generalisability of our findings. Although the response rate to the reliability study was low, our sample had a similar gender and ethnic composition to that of the 2011 Census findings [29] for the local borough of Charnwood from which our sample was recruited. There was a small difference in age distribution, with a lower proportion of those aged under 30 and a higher proportion of those aged over 45 represented in our sample. In the validity study additional strategies were necessary to recruit sufficient participants, resulting in the inclusion of staff and students from within the University and local workplaces that may have reduced the overall representativeness of the study population.

Adherence with the validity study protocol was generally very high, although a small number of participants did not wear the accelerometer for sufficient time on all seven days of the measurement period. We were able to adjust for this in the analysis using standard procedures. Some participants were excluded from the analysis because they did not meet the strict accelerometer wear time criteria or did not complete the survey with two days of their final day of wearing the accelerometer. There were no significant differences in key characteristics between participants included and excluded for these reasons, except that a higher proportion of those excluded were aged 30–44 (data not shown). Given the low number of participants in the validity study, and the inclusion of only a small number of adults aged 65 and over, further investigation may be required to assess the measurement properties of the TPAQ in different age groups and in different contexts.

Finally, it is difficult to distinguish between different forms of activity using conventional methods of processing accelerometer data, and it was therefore not possible to assess the validity of self-reported estimates of time spent in the specific behaviours of walking and cycling in this study. To derive objective measures of mode-specific travel times in this sample requires a combination of accelerometer and GPS data that will be pursued in subsequent analyses.

## Conclusions

This study reports on the development and selected measurement properties of a new comprehensive instrument (the TPAQ) for use in the assessment of physical activity and travel behaviours. Overall, the reliability and validity of the TPAQ for measuring total physical activity, and specific domains of physical activity including walking and cycling for different purposes, are comparable to those of existing physical activity questionnaires of similar length currently being used. The TPAQ may therefore provide an alternative instrument suitable for wider use.
